# Nb_2_C and Nb_2_CO_2_ MXenes as Anodes in Li-Ion Batteries: A Comparative Study by First-Principles Calculations

**DOI:** 10.1021/acsomega.4c03603

**Published:** 2024-06-21

**Authors:** Raúl Santoy-Flores, Héctor Noe Fernández-Escamilla, José Israel Páez-Ornelas, Eduardo G. Perez-Tijerina, Jonathan Guerrero-Sánchez, Rodrigo Ponce-Pérez, Noboru Takeuchi, Ma. Guadalupe Moreno-Armenta

**Affiliations:** † Facultad de Ciencias Físico-Matemáticas, Universidad Autónoma de Nuevo León, Código Postal, San Nicolás de los Garza, Nuevo León 66451, Mexico; ‡ Centro de Nanociencias y Nanotecnología, Universidad Nacional Autónoma de México, Apartado Postal 14, Código Postal, Ensenada, Baja California 22800, Mexico

## Abstract

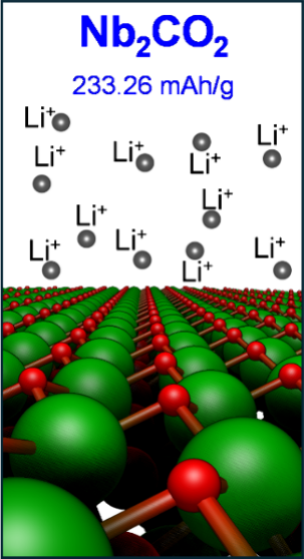

The new generation of Li-ion batteries is based on integrating 2D materials into the electrodes to increase the energy density while reducing the charging time and size. The two-dimensional transition metal carbide or nitride (MXene) materials offer ideal electronic properties, such as metallic behavior, low energy barriers for Li-ion diffusion, and structural stability. This study focuses on Nb_2_C and Nb_2_CO_2_ MXenes, which have shown promising Li-storage capacity, especially the oxidized phase. By using density functional theory (DFT) and thermodynamic criteria, we studied the Li intercalation process in both MXenes. The results show that the Li intercalation process in the oxidized phase is more stable. Also, the Li diffusion barriers are 35 and 250 meV for the bare and oxidized phase, due to the strong interaction between Li ions and O functional groups. Nb_2_C and Nb_2_CO_2_ MXenes deliver a maximum gravimetric theoretical capacity of 275 and 233.26 mA h/g, respectively, with a stable performance.

## Introduction

Nowadays, technological advances demand devices capable of efficient storage and transport. The devices based on electrochemical mechanisms, such as batteries, are the most common for this purpose since they are easy to transport, store great energy capacity, and are compatible with our electronic devices. There are three reported electrochemical mechanisms that store energy (ions), known as pseudocapacitance:^1−3^ (1) under-potential pseudocapacitance, (2) redox pseudocapacitance, and (3) intercalation pseudocapacitance, each has advantages and disadvantages, but more benefits have been found for the pseudocapacitance of ion intercalation in crystalline electrodes. Moreover, recent investigation affirms that the intercalation pseudocapacitance, which employs crystalline structures as electrode materials, provides a higher energy density than under-potential and redox pseudocapacitance.^3^ Notably, low-dimensional materials as electrodes are being proposed as the next generation of Li-ion batteries due to the high surface reactivity because it improves the adsorption of adatoms and molecules.^4^ Besides, organic 2D materials are emerging candidates for Li-ion batteries.^5^ On the other hand, the reuse of electrodes from retired Li-ion batteries is an option for recycling components since electrodes can be employed in Na-ion batteries.^6^

The MXenes are a new family of two-dimensional layered transition metal carbides or nitrides with the general formula M_*n*+1_X_*n*_T_*y*_ (*n* = 1, 2, 3), where M is the transition metal, X is carbon or nitrogen atoms, and T_*y*_ may be different functional groups (O, F, OH, Cl), even the H atom, which imparts hydrophilicity to their surfaces and tunes the electronic properties. These materials are ideal for applications including energy storage,^7^ electromagnetic interference shielding,^8^ reinforcement for composites,^9^ water purification and gas and biosensors,^10,11^ lubrication,^12^ and photo-,^13^ electro-,^14^ and chemical catalysis.^15^ Moreover, electronic,^16^ optical,^17^ plasmonic,^18^ and thermoelectric properties have also been observed.^19^ Since the synthesis of the Ti_3_C_2_ MXene in 2011,^20,21^ dozens of MXenes have been predicted, and their applicability has been studied theoretically.^22^ Among these materials, the Nb_2_C synthesized in 2013 by Naguib et al.^23^ exhibits a good ability to handle high cycling rates (10C). Also, it can be used as a promising material for energy storage devices such as Li-ion batteries. The Nb_2_C and Nb_2_CT_*y*_ show excellent conductivity; Nb_2_C is reported as superconducting with a critical temperature of *T*_c_ = 12.5 K, which is the highest critical temperature among the 2D MXenes;^24^ furthermore, previous calculations demonstrate Dirac points at Nb_2_C in the vicinity of the Fermi level.^25^ Moreover, Hu et al.^26^ stated that the most stable structure for terminated MXene is the fully O-terminated configuration, being the most favorable in terms of capacity. Although the weight is a crucial fact in Li storage to achieve a high gravimetric capacitance, the Nb_2_CT_*y*_ capacity exceeds that of Ti_2_CT_*y*_ in some cases (180 and 110 mA h/g, respectively, at 1C).^23^

The low energy barriers for Li-ion diffusion on the pristine MXene suggest improved energy storage performance. At the same time, the functionalized MXenes show a high energy barrier, making them less favorable for Li-ion diffusion. Previous reports on Nb_2_C and Nb_2_CO_2_ have provided primary results for improving Li-ion battery electrodes.^27,28^ Zhu et al.^29^ reported specific capacities of 305 and 292 mA h/g for Nb_2_C and Nb_2_CO_2_, respectively,^29^ with a maximal Li concentration of 2.25 atoms per unit cell and 2.5 atoms per unit cell for Nb_2_C and Nb_2_CO_2_ respectively, being the Li adsorption (lithiation) on both sides. Since recent experimental and theoretical reports suggest a promising future for MXenes toward Li-ion batteries, in this work, the Li storage capacity on Nb_2_C and Nb_2_CO_2_ was studied by density functional theory (DFT) calculations. The Li adsorption and diffusion mechanism was investigated. Subsequently, the lithiation process is studied by systematically increasing the Li coverage to reach higher energy density levels. We describe the lithiation mechanism by structural model analysis, energy stability, and electrochemical properties to compare the Nb_2_C and its oxidized phase.

## Methodology

The Li intercalation process onto Nb_2_C and Nb_2_CO_2_ MXenes was investigated by first-principles calculations. All calculations were performed in the periodic DFT framework as implemented in the PWscf code of the Quantum Espresso package.^30,31^ The exchange-correlation energy was described employing the Generalized Gradient Approximation (GGA) with Perdew–Burke–Ernzerhof (PBE) parametrization.^32^ The electron-ion interactions were treated employing Vanderbilt ultrasoft pseudopotentials^33^ with 550 eV as the energy cutoff. The van der Waals interactions were considered by the dispersion correction functional Grimme-D3.^34^ The supercell method is employed to simulate MXene; each supercell is formed by a vacuum space larger than 20 Å to avoid interactions between periodic slabs. Also, each slab is formed by a monolayer of Nb_2_C or Nb_2_CO_2_ in a 3 × 3 periodicity. In the geometry optimization, convergence is achieved when all force components are smaller than 0.026 eV/Å, and the total energy differences must be less than 1 × 10^–4^ eV. The Brillouin zone was sampled with the special *k*-point scheme of Monkhorst–Pack^35^ with a grid of 4 × 4 × 1. To study the minimum energy pathway (MEP) for the Li diffusion over the surface we employed the Climbing image-nudged elastic band (CI-NEB) method^36^ with 11 intermediate images.

## Results and Discussion

### Nb_2_C and Nb_2_CO_2_ MXenes Structure

**Figure 1:**
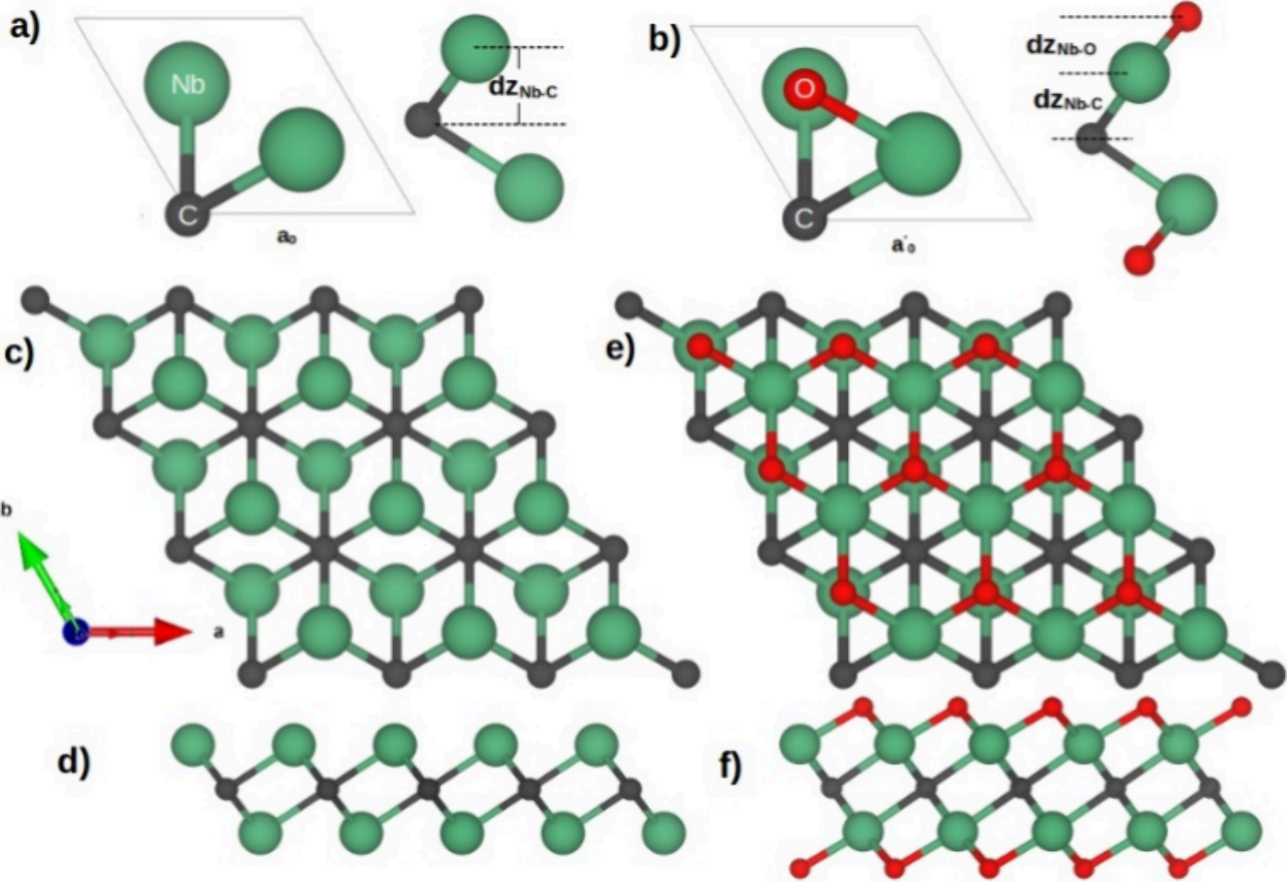
Atomistic models for the Nb_2_C and Nb_2_CO_2_ MXenes. (a and b) are for the unit cell of the Nb_2_C and Nb_2_CO_2_, respectively. (c and d) top and side view of the 3 × 3 supercell of Nb_2_C. (e and f) top and side view of the 3 × 3 supercell Nb_2_CO_2_. The green, gray, and red spheres represent the Nb, C, and O atoms, respectively.

The atomistic representation of the Nb_2_C and Nb_2_CO_2_ MXenes unit cells are depicted in Figure 1a, b, respectively. Also, their corresponding top and side views of the 3 × 3 supercell are in Figure 1c, d for Nb_2_C and in Figure 1e, f for Nb_2_CO_2_. The calculated cell parameter for bare Nb_2_C is 3.11 Å, with a C–Nb bond distance of 2.15 Å and an interlayer distance of C–Nb of 1.19 Å. Conversely, oxidized Nb_2_C has a cell parameter of 3.13 Å with Nb–O and Nb–C bond distances of 2.10 and 2.20 Å, respectively. The calculated lattice parameter for the oxidized phase agrees with the experimental reports of the Nb_2_CT_*x*_ MXenes, which report a lattice parameter of 3.13–3.16 Å^37^; therefore, no additional corrections, such as Hubbard (DFT+*U*), are needed. Also, the interlayer distance between Nb and O atomic layers is 1.07 Å, while the interlayer distance for Nb–C is 1.25 Å. Table 1 summarizes the structural parameters of both MXenes. The phonon spectra for both Nb_2_C and Nb_2_CO_2_ have previously been reported, where non-negative frequencies were observed denoting their dynamic stability.^38^

**Table 1:** Calculated Lattice Parameters, Bond Lengths, MXene Thickness (d*z*), and Interplanar Distances Nb–C (d*z*_Nb–C_) and Nb–O (d*z*_Nb–O_) for Both Nb-Based MXenes^a^

MXene	*a* (Å)	C–Nb (Å)	Nb–O (Å)	d*z* (Å)	d*z*_Nb–C_ (Å)	d*z*_Nb–O_ (Å)
Nb_2_C	3.11	2.15		2.37	1.19	
Nb_2_CO_2_	3.13	2.20	2.10	4.63	1.25	1.07

a Lattice parameter C–Nb: bond distance C–Nb Nb–O: bond distance Nb–O.

**Figure 2:**
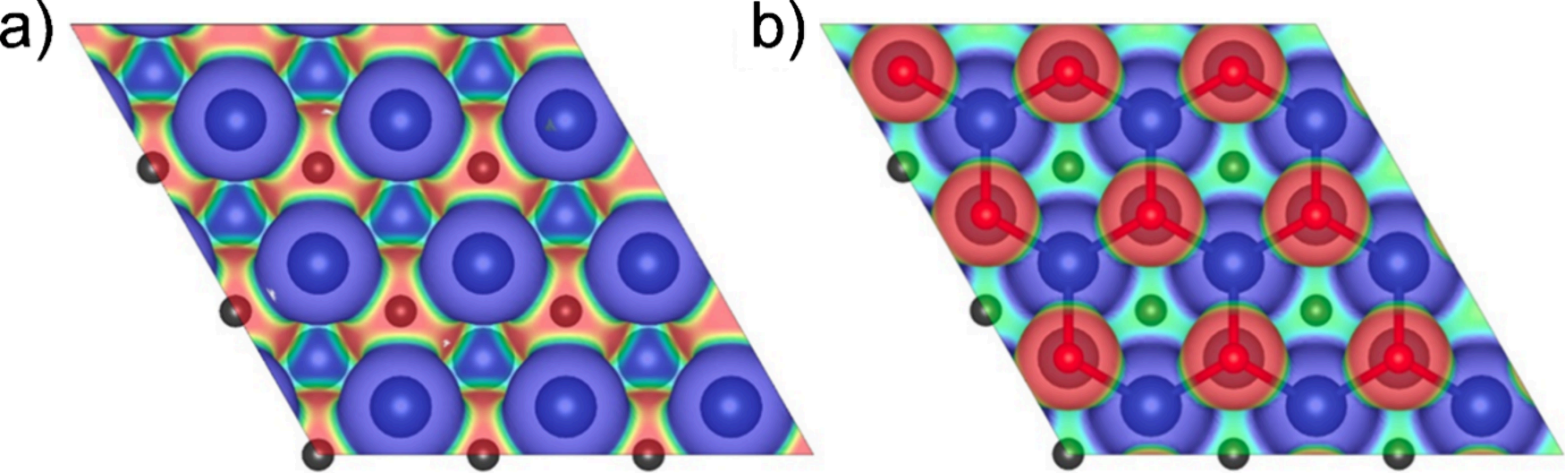
Electrostatic potential isosurfaces (with an isovalue of 0.05 a.u.) for (a) Nb_2_C and (b) Nb_2_CO_2_ in the RGB color scheme. Regions of positive, neutral, and negative potentials are in red, green, and blue color, respectively.

We also calculated the electrostatic potential isosurfaces (EPI) for both MXenes to get insights into the electrostatic nature of the interactions that lead to Li adsorption on the different positions. The isosurfaces with isovalues of 0.05 a.u. for Nb_2_C and Nb_2_CO_2_ are shown in Figure 2a, b, respectively. The isosurfaces are represented in the RGB scheme, where the blue color represents a positive electrostatic potential, and the red color is for a negative electrostatic potential. It can be noted that on both MXenes, the Nb atoms present positive electrostatic potential, as indicated by the blue isosurfaces surrounding them. Moreover, the C atoms show negative potential on Nb_2_C due to the light-red color. Meanwhile, at Nb_2_CO_2_, the C atoms are surrounded by a green surface denoting neutral potential, and it can be attributed to the presence of the O, which presents a high negative potential due to its high electronegativity. The negative electrostatic potential at the MXenes can be a highly reactive site by attracting the Li atoms. Therefore, a strong interaction will occur when the Li atoms are placed close to the oxygen.

**Figure 3:**
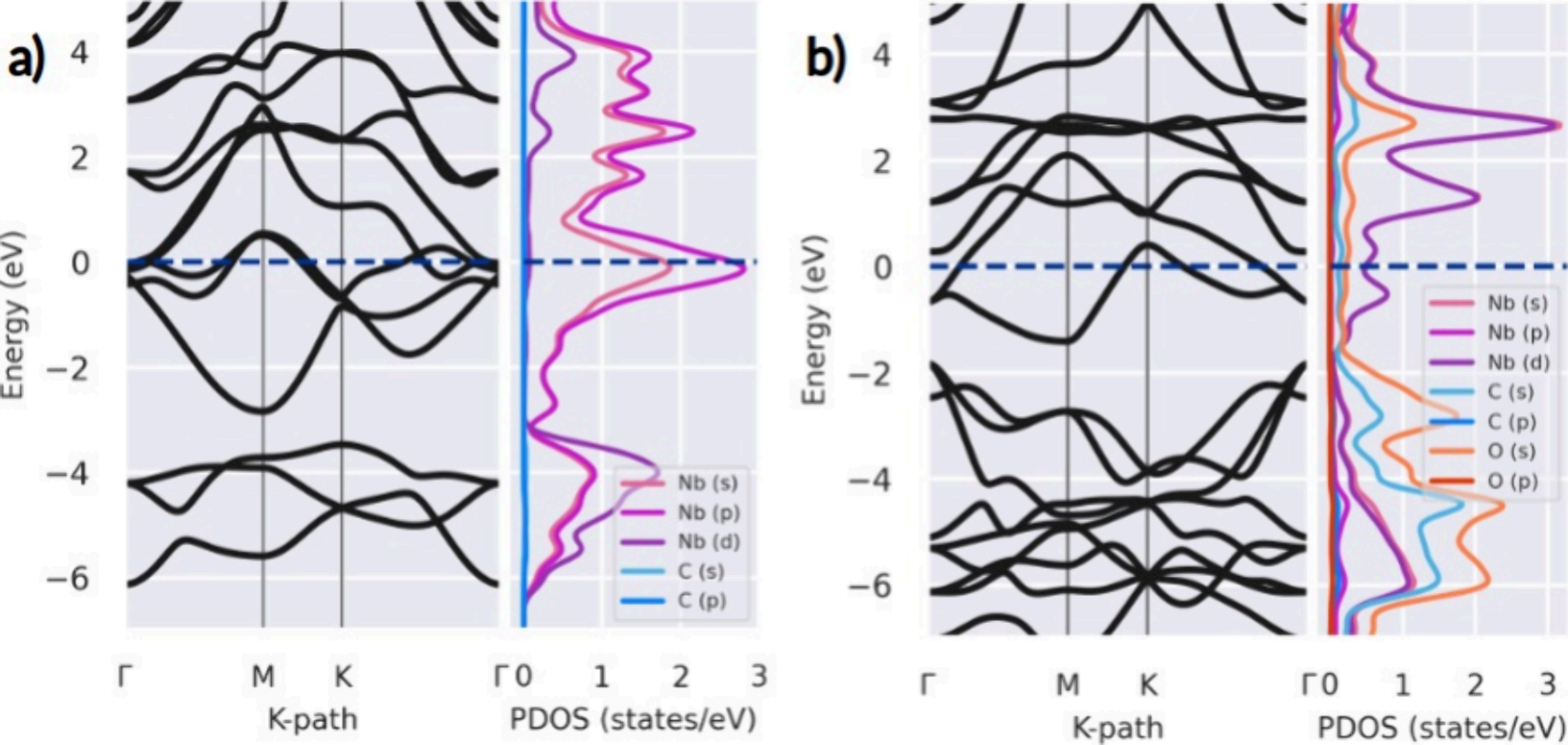
Electronic band structure along the Γ–*M*–*K*–Γ path and their corresponding projected density of states for (a) Nb_2_C and (b) Nb_2_CO_2_ MXenes.

The electronic properties of MXenes are investigated by calculating the projected density of states (PDOS) and the band plot diagram along the Γ–*M*–*K*–Γ path. In all cases, the energy reference is set at the Fermi level. The results are listed in Figure 3. Our results are consistent with Yang and Ting,^25^ showing metallic behavior for Nb_2_C and Nb_2_CO_2_ favorable for good anode performance. Moreover, the PDOS shows the electronic participation from each atom, which suggests that the Nb-p orbitals mainly contribute to the density of states at the Fermi level for bare MXene, followed by the Nb-d contributions. Meanwhile, for oxidized MXene, the Nb-d and Nb-p orbitals have equivalent contributions around the Fermi level, which are attributed to the oxidation of the surface. Besides, it is noticed that the main contribution to the DOS at energies below the Fermi level (from −2 to −6 eV) comes from the oxygen atoms in the oxidized MXene and Nd atoms for the bare Nb_2_C.

### Li Intercalation and Diffusion

**Figure 4:**
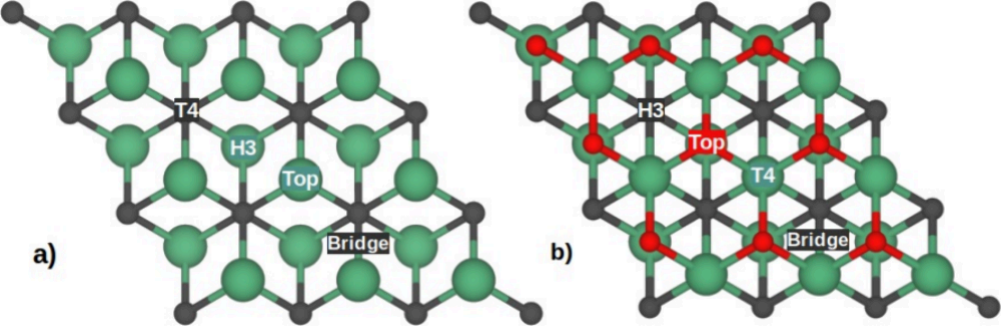
Four HSS tested during the adsorption of Li on (a) Nb_2_C and (b) Nb_2_CO_2_ MXenes. The green, gray, and red spheres represent the Nb, C, and O atoms, respectively.

Previous reports have demonstrated that the Li intercalation process is carried out on the MXene surface.^39^ Therefore, we evaluated the Li intercalation process by considering four high symmetry sites (HSS) on the surface. In both MXenes, we took the most exposed layer as a reference. The top site occurs when a Li atom is placed on top of the atom of the most exposed layer. The T4 site occurs if the Li atom is placed on top of the atoms of the second most exposed layer. Meanwhile, the H3 site is on top of the third most exposed layer. Finally, the Bridge site is the middle point between the two atoms of the first layer. Figure 4 shows the four HSS values for both MXenes considered in this work.

We also calculated the adsorption energies (*E*_ads_) for the Li adsorption onto the surface following the following equation:
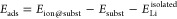
1where *E*_ion@subst_ is the total energy of MXene with Li adsorbed on it, *E*_subst_ is the total energy of the MXene without interaction with the Li atoms, and *E*_Li_^isolated^ is the total energy of an isolated Li atom simulated in an empty box of 20 Å of length. With this definition, the negative *E*_ads_ values denote favorable adsorption and positive values denote unfavorable adsorption. The calculated *E*_ads_ values are summarized in Table 2. Our results display that for the Nb_2_C MXene, the most favorable site is the T4, which corresponds to placing the atom on top of the C atoms, the zone with the most negative electrostatic potential, as shown in Figure 2a, followed by the H3, Bridge, and Top (T4 < H3 < bridge < top). Also, the H3 site is the most stable configuration for Li adsorption onto the Nb_2_CO_2_ MXene. This site is a neutral region surrounded by three oxygen atoms (negative region) interacting with Li, performing a three-fold coordination. It is the area furthest from the positive region formed by the Nd atoms, as shown in Figure 2b, enhancing their stability. Besides, the Bridge site is unstable and tends to move to the H3 site. The T4 and Top sites are less stable (H3 = bridge < T4 < top). Also, note that for the Nb_2_CO_2_, the MXene–Li interaction is stronger. This fact is attributed to the negative electrostatic potential of the O atoms, favoring stronger Li adsorption, as shown in the previous section.

**Table 2:** Calculated Energy Adsorption *E*_ads_ (in eV) for Li at Nb_2_C and Nb_2_CO_2_

	*E*_ads_ (eV)	
site	Nb_2_C	Nb_2_CO_2_
top	–2.23	–2.97
bridge	–2.34	–3.51
T4	–2.37	–3.26
H3	–2.35	–3.51

**Figure 5:**
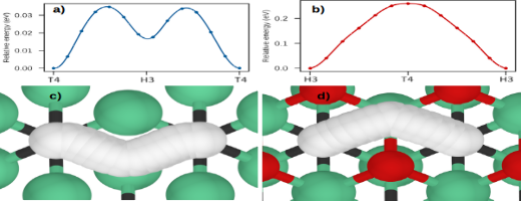
Energy barriers to Li-ion diffusion on (a) Nb_2_C and (b) Nb_2_CO_2_. Li-ion atomic trajectories are shown in (c and d). The presented MEP was calculated by using the CI-NEB method. The green, gray, red, and white spheres represent the Nb, C, and Li atoms, respectively.

An essential feature of the electrodes is their capacity to release ions, which involves low diffusion energy barriers. We have used the CI-NEB method to study Li diffusion along the surface of Nb_2_C and Nb_2_CO_2_ MXenes. Figure 5 shows the computed MEP for Li diffusion. Figure 5a, c corresponds to the MEP for diffusion along the Nb_2_C surface and their corresponding atomic representation. The diffusion happens from a T4 site to another equivalent position. The bridge site is the energy barrier required to diffuse the Li adatom with a value of 35 meV, the H3 site is a metastable site with an energy 18 meV less than T4, also, to move the Li from H3 site to T4, an energy of 17 meV is required. About the Nb_2_CO_2_ MXene, Li diffusion is carried out from the H3 site to an equivalent position. Our results demonstrate that oxidized surfaces modify the diffusion pathway. Figure 5b, d shows the MEP and their atomic description, respectively. In this case, the T4 site is the energy barrier present in the Li diffusion with a value of 250 meV, which is a consequence of the strong interaction of the functional group with the Li atom. Although oxidized MXene provides energy barriers that are 1 order of magnitude larger than bare MXene, the activation energy is lower than that of some Ti-based functionalized MXenes.^3^

### Lithiation Mechanism

**Figure 6:**
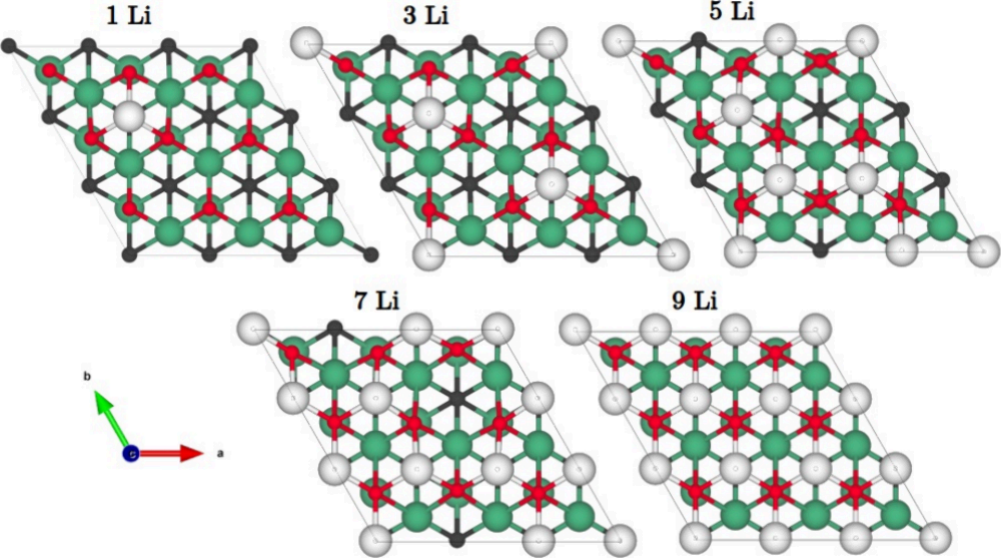
Atomistic models for the Li intercalation mechanism from 1 to 9 Li. The green, gray, red, and white spheres represent the Nb, C, O, and Li atoms, respectively.

Once we investigated the most favorable site for the Li intercalation and their energy barriers to diffuse the atom across the surface, we systematically added more Li atoms to the MXenes to saturate the surface. Note that we considered only one side of the MXenes. Figure 6 shows the intercalation of Li ions up to a full monolayer. We focus on the oxidized MXene since bare Nb_2_C shows similar behavior. Once one Li is incorporated, the following atoms are placed around, forming a line (3Li model). After that, subsequent Li atoms occupy the H3 sites closer to the line formed by Li (5Li models). Once 7Li atoms are incorporated into the MXene surface, it is noticed that Li forms hexagonal patterns with a hollow in the center, which is covered when full ML is formed. Our findings show that the Li intercalation mechanism is ordered and follows a pattern up to a full Li ML.

**Figure 7:**
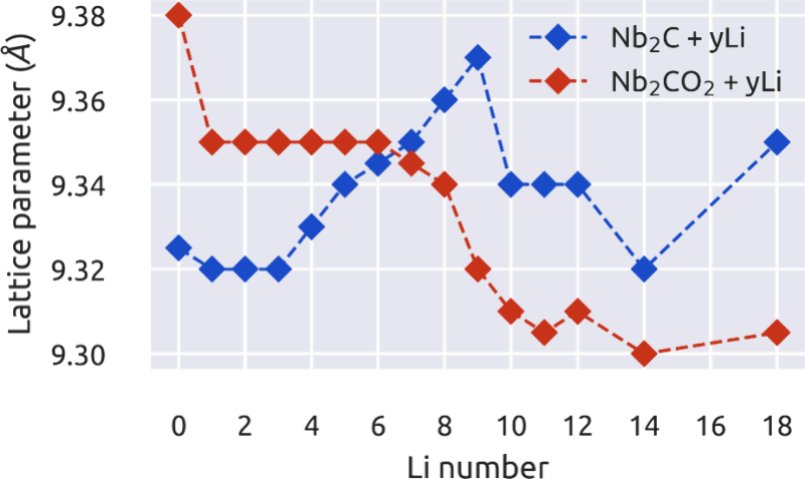
Evolution of the cell parameter as a function of the Li content in the surface of Nb_2_C and Nb_2_CO_2_ MXenes. The blue line represents the Li incorporation on Nb_2_C MXene, while the red line is for the lithiation process onto Nb_2_CO_2_ MXene.

**Figure 8:**
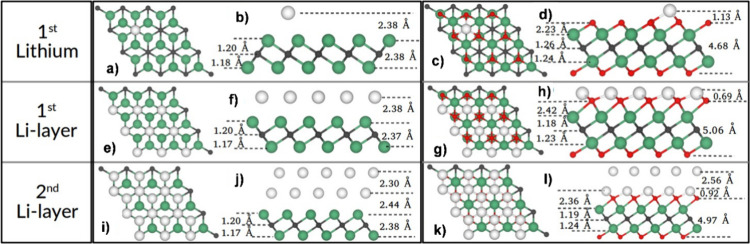
Adsorption of a single Li atom and the Lithiation process to achieve a single and double Li-ML over the Nb_2_C and Nb_2_CO_2_ phases. (a–d) adsorption of a Li atom, (e–h) formation of a Li-ML by the adsorption of nine Li atoms, and (i–l) formation of a double-stacked Li-ML. The green, gray, red, and white spheres represent the Nb, C, O, and Li atoms.

The evolution of the cell parameters as a function of the number of Li molecules present in the structure is depicted in Figure 7. About the Nb_2_C compound, the first Li atoms occupy the T4 sites, as shown in Figure 8a,b, with an interlayer distance of 2.38 Å for the bottom Nb monolayer. Also, we notice an expansion in the cell parameter (see the blue line in Figure 7). The MXene without Li has a cell parameter of 9.33 Å for the 3 × 3 supercell. After the fourth Li adsorbed, the Nb_2_C lattice parameter expands up to 9.37 Å when nine Li atoms are placed onto the surface; this corresponds with the formation of a full Li monolayer (ML) onto the surface, see Figure 8e,f. After that, a second monolayer is placed over the first layer of Li atoms (Figure 8i,j). The second Li layer is accompanied by a reduction in the cell parameter, up to 9.35 Å.

On the other hand, the Nb_2_CO_2_ structure with a cell parameter of 9.39 Å for the 3 × 3 supercell suffers a contraction in the cell parameter as the Li content increases. See the red line in Figure 7. The first Li adsorbed in the H3 site (Figure 8c, d) reduces the Nb_2_CO_2_ lattice parameter to 9.35 Å, and it remains unchanged until the incorporation of the seventh Li, where it continues decreasing with an interlayer distance of 0.69 Å between the Li and O ML. The cell parameter is contracted to 9.32 Å when the full Li monolayer is formed; see Figure 8g, h. Besides, the MXene parameter is practically constant when the second full monolayer is included. Also, the interlayer distance between Li monolayers is 2.30 and 2.56 Å for the Nb_2_C and Nb_2_CO_2_ MXenes, respectively, as shown in Figure 8j,l.

### Lithiation Energy Stability Analysis

**Figure 9:**
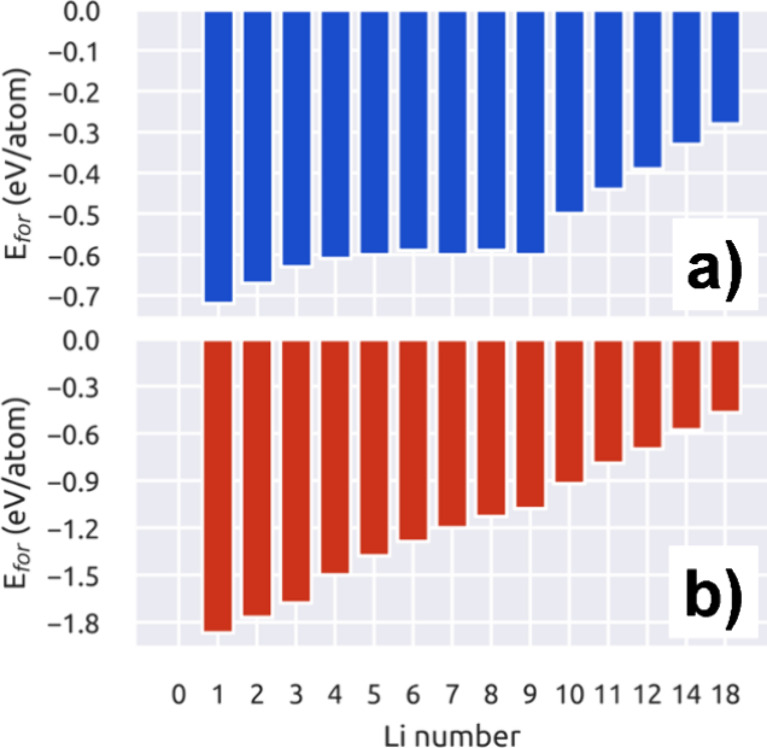
Formation energies (in eV/atom) vs Li content for the Li intercalation process on (a) Nb_2_C and (b) Nb_2_CO_2_ MXenes.

Once the lithiation mechanism was described, we analyzed the thermodynamic stability of the lithiated systems using the formation energies formalism.^39,40^ In our case, the formation energy can be adapted to our systems as follows:
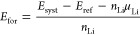
2where *E*_syst_ is the total energy of the system at hand, *E*_ref_ is the total energy of the MXene without interaction with Li atoms, *n*_Li_ is the number of Li atoms adsorbed in the system, and μ_Li_ is the Li chemical potential defined as  with *E*_Li_^bulk^ as the total energy of the body-centered cube (BCC) unit cell and *n*_Li_^bulk^ as the number of atoms present in the unit cell. This equation shows negative values for a favorable structure, while positive values suggest thermodynamic instability. The calculated *E*_for_ of all models is shown in Figure 9. The upper and lower panels are for Nb_2_C and Nb_2_CO_2_ MXenes, respectively. In both cases, it is noticed that the lithiation process is favorable. About the Nb_2_C MXene, the first Li atom has a formation energy value of −0.72 eV/atom, see Figure 9a, this value slightly increases when three Li are adsorbed onto the surface. Also, the *E*_for_ remains constant up to form the full Li coverage; after that, for the Li atoms that form the second monolayer, the *E*_for_ increases as the number of Li atoms increases. However, the complete second Li monolayer remains stable with an *E*_for_ of −0.28 eV/atom.

About the Nb_2_CO_2_ MXene, the first Li adsorbed has an *E*_for_ = −1.87 eV/atom, as depicted in Figure 9b, the formation energy values start to decrease as the number of Li adsorbed onto the surface increases, and the full Li ML has an *E*_for_ = −1.08 eV/atom; besides, the formation of two full Li monolayers has an *E*_for_ = −0.47 eV/atom. Although the formation of two Li MLs is stable in both MXenes, it is noticed that the oxidized phase has lower formation energy values. This result suggests that the lithiation process onto the surface of Nb_2_CO_2_ MXene is more stable than the same process on the Nb_2_C MXene surface.

### Charge Distribution

We perform a Bader charge analysis to investigate the structure’s charge distribution. The C atoms from Nb_2_C MXene are 6-fold coordinated with Nb atoms. Also, the C atoms accept 1.84e (0.30e/bond), while the Nb atoms, which are 3-fold coordinated, donate 0.90e to C atoms (0.30e/bond). Similarly, the C atoms from Nb_2_CO_2_ accept 0.30e from their bonds. Also, O atoms take from the three neighboring Nb atoms 1.14e (0.38e/bond). Once the Li atoms are placed onto the Nb atoms in the Nb_2_C MXene, each Li shares 0.81e with the substrate, while in the case of the Nb_2_CO_2_, each Li donates 0.86e.

**Figure 10:**
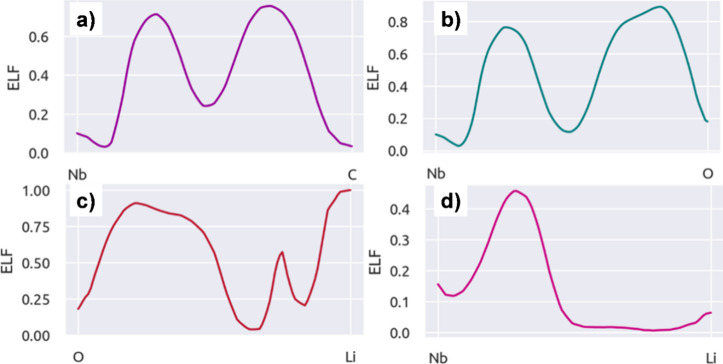
Line profiles along the bonds present in the Nb_2_C and Nb_2_CO_2_. (a) Nb–C, (b) Nb–O, (c) O–Li, and (d) Nb–Li.

To investigate the nature of the bonds present in our systems, we perform electron localization function (ELF) line profiles. The results are depicted in Figure 10. Note that the electron population’s decay around the atoms’ core is a consequence of the use of pseudopotentials. The line profiles for the Nb–C and Nb–O interactions are given in Figure 10a,b, respectively. In both cases, an ionic character is observed. Regarding the Nb–Li interaction, the poor electron population along the bond suggests a weak interaction between them, as shown in Figure 10d, which explains the lower energy barriers present in the MEP for Li diffusion across the surface. In addition, for the interaction between the atoms from the Nb_2_CO_2_ MXene and the Li atoms, see Figure 10c, we notice electron population along the bond, which suggests a strong interaction between them and is related to the large energy barrier in the MEP.

Usually, in the Li intercalation process an expansion in the cell parameter is observed as the number of Li increases, the expansion is associated with the ion size and the electrostatic repulsion between them.^41^ The same behavior is observed in the Nb_2_C MXene, and the ELF line profiles show weak interactions between Nb and Li atoms which enhance the electrostatic repulsion between Li increasing the cell parameter. On the other hand, some MXenes have shown the opposite behavior, where a reduction in the cell parameter is observed, such as Ti_2_CO_2._^41^ This effect is associated with changes in the surface’s charge distribution because of the functional group. About Nb_2_CO_2_ MXene, Li is placed between oxygen atoms, increasing the interaction between the MXene and Li as demonstrated by the ELF profiles. This rearrangement of charges reduces the electrostatic repulsion between Li reducing the cell parameter.

### Electrochemical Properties

To evaluate the electrochemical capacities of the MXenes as anodes in Li-ion batteries, we calculated the average electrode potential (*V*) as a function of the theoretical gravimetric capacity (*Q*) following the next reaction for Nb_2_C and Nb_2_CO_2_ MXenes, respectively:
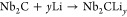
3
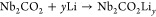
4where *y* is the concentration of Li atoms per unit cell, the electrode potential versus Li/Li^+^ counter electrode was estimated to find the electrodes’ maximum theoretical gravimetric capacity (QMAX). If *y*Li are adsorbed onto the MXene with *n*–*y* Li atoms to form a new structure, the average electrode potential can be calculated following the next expression:^42^
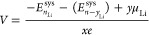
5where *E*_*n*_Li__^sys^ is the total energy of the MXene with *n*Li adsorbed on it, *E*_*n*–*y*_Li__^sys^ is the total energy of the MXene with (*n*–*y*)Li adsorbed on the surface, μ_Li_ is the chemical potential of Li atoms, and *e* is the elemental electron charge. Also, the theoretical gravimetric capacity can be calculated as follows:
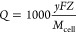
6where *Z* is the valence of Li atoms (*Z* = 1), *F* is the Faraday constant (26.81 A h/mol), and *M*_cell_ is the atomic weight of the electrode material.

**Figure 11:**
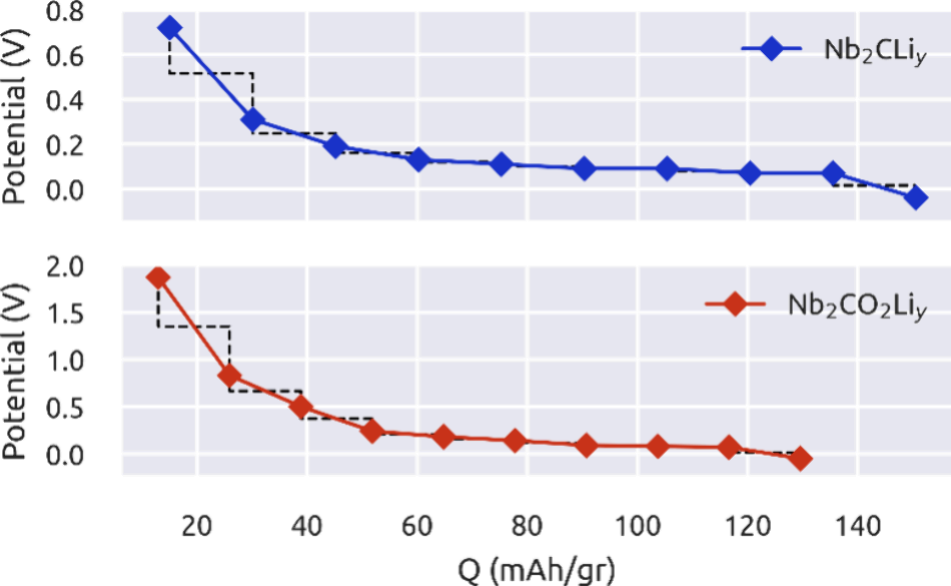
Potential vs *Q* plots for the Li intercalation process in the Nb_2_C and Nb_2_CO_2_ electrodes.

Figure 11 summarizes the results. Note that both MXenes provide a stable performance. Oxidized MXene has the highest voltage of 1.87 V, while the highest value for Nb_2_C is 0.72 V at capacities of 12.96 and 15.06 mA h/g, respectively. Both MXenes exhibit lower voltages around ≈0.1 V at 116.63 and 135.50 mA h/g for the Nb_2_CO_2_ and Nb_2_C MXenes, respectively.

It is worth mentioning that our calculations consider only one side of the MXenes. However, the Li intercalation process occurs on both sides of the material. In our previous work, we noticed that the most stable configuration occurs when interaction preserves the inversion symmetry by considering the two faces of the MXene, implying that both surfaces are equivalent.^39^Table 3 shows the maximum gravimetric capacity for both MXenes considering the Li intercalation process on one or two sides of the MXene. Nb_2_C delivers a gravimetric capacity of 135.50 mA h/g if The Li intercalation process occurs on one side of the electrode and has a maximum gravimetric capacity of 275 mA h/g if the intercalation process is carried out on both sides. On the other hand, the Nb_2_CO_2_ MXene delivers a gravimetric capacity of 116.63 mA h/g if the process occurs in one side of the MXene and a maximum gravimetric capacity of 233.26 mA h/g if the Li intercalation process occurs in both sides.

**Table 3:** Theoretical Maximum Gravimetric Capacity for Both Nb-Based MXenes Considering One Side or Two Sides

maximum gravimetric capacity for Li storage		
MXene	model	*Q*_MAX_ (mA h/g)
Nb_2_C	one side	135.50
	two sides	275.00
Nb_2_CO_2_	one side	116.63
	two sides	233.26

Our results agree with the experimental results obtained by Naguib et al.^23^ where they found gravimetric capacities of 170 mA h/g for the Nb_2_CT_*x*_ MXene. The experimental value is closer to the result obtained for the Nb_2_CO_2_ (233.26 mA h/g) than for Nb_2_C MXene (275 mA h/g) because in the experiment the surfaces are decorated with functional groups. However, experimental results provide lower values in comparison with theoretical results. This is attributed to the fact that the surface is not only functionalized with O atoms but there is also the presence of OH and F groups.

Finally, Table 4 shows the theoretical gravimetric capacity and energy barriers for different MXenes and other 2D materials theoretically investigated by their implementation as an anode in Li-ion batteries. Note that bare Nb_2_C has activation energies of the order of Ti_4_C_3_, Ti_2_Ta_2_C_3_, and Ti_2_C MXenes, while Nb_2_CO_2_ has energy barriers lower than Ti-based MXenes functionalized but large in comparison with V_2_CO_2_. Besides, the theoretical gravimetric capacity of the Nb-based MXenes is similar to that of the Ti-based counterparts, but V-based MXenes exhibit better capacities. On the other hand, the activation energies observed in SiC_3_N_3_ are slightly larger in comparison, and the observed in g-CN and BC_3_N_3_ compounds are very large to be employed in Li-ion batteries.

**Table 4:** Theoretical Gravimetric Capacity and Activation Energies of Different 2D Materials Employed in Li-Ion Batteries

material	*Q* (mA h/g)	activation energy (meV)	ref
Nb_2_C	275.00	35	this work
Nb_2_CO_2_	233.26	250	this work
Ti_4_C_3_	305.79	33	(39)
Ti_2_Ta_2_C_3_	142.86	31	(39)
Ti_2_C	497.00	20	(43) and (44)
Ti_2_NO_2_	378	249	(45)
Ti_2_NF_2_	90.00	278	(45)
Ti_2_N(OH)_2_	no adsorption		(45)
Ti_3_C_2_O_2_	268.53	411	(46)
V_2_CO_2_	335.50	180	(43)
SiC_3_N_3_	253.00	430	(47)
g-CN		2940	(48)
BC_3_N_3_		1070	(48)

## Conclusions

By DFT calculations, we investigated the Li intercalation process on Nb_2_C and Nb_2_CO_2_ MXenes. The T4 and H3 sites are the most stable configurations for Li adsorption onto the surface of the Nb_2_C and Nb_2_CO_2_ MXenes, respectively. The minimum energy pathway for the diffusion of Li atoms over both phases shows an associated energy barrier of 35 meV for Nb_2_C and 250 meV for Nb_2_CO_2_, and the low energy barrier for bare MXene is attributed to the low interaction with the Nb atoms of the electrode. Also, the energy barriers for the oxidized phase are lower than some Ti-based functionalized MXenes. As the number of Li increases, the Nb_2_C phase experiences an expansion in the cell parameter, while Nb_2_CO_2_ suffers a contraction. However, in both cases, the deposit of a full Li monolayer is thermodynamically stable, and the presence of two Li MLs remains stable. The maximum theoretical gravimetric capacities are 275 and 233.26 mA h/g for Nb_2_C and Nb_2_CO_2_ MXenes, respectively, with stable performance. Our calculated capacities are slightly above the experimental values; however, this can be attributed to a mixed phase of O, OH, and F functional groups over the surface. Our finding provides insights into the Li intercalation process and the expansions and contractions that suffer the electrodes in the charge and discharge process, demonstrating that the Nb-based MXenes are good candidates to be implemented in Li-ion batteries.

## Supporting Information Available

Band structure files for Nb_2_C and Nb_2_CO_2_ MXenes in .dat extension (ZIP)
